# Elevated Troponin I in the Absence of Coronary Artery Disease: A Case Report With Review of Literature

**DOI:** 10.14740/jocmr2280w

**Published:** 2015-08-23

**Authors:** Sabrina Arshed, Hong Xiu Luo, Shoaib Zafar, Kalyani Regeti, Nilma Malik, Mahmood Alam, Abdalla Yousif

**Affiliations:** a Raritan Bay Medical Center, Internal Medicine Residency Program, 530 New Brunswick Avenue, Perth Amboy, NJ 08861, USA

**Keywords:** Troponin, NSTEMI, Idiopathic, Myocardial injury, Coronary artery disease, Cardiac biomarkers

## Abstract

Cardiac troponins are the most sensitive and specific markers of myocardial injury. In fact, the Joint European Society of Cardiology/American College of Cardiology committee for the redefinition of myocardial infarction (MI) states that troponins are the preferred cardiac marker for detecting myocardial injury. For the aforementioned reasons, troponin levels are routinely ordered for patients presenting to the emergency department with chest pain, dyspnea, syncope, or any other possible presentations of MI. While troponin levels do reflect the extent of myocardial damage, they do not necessarily indicate myocardial ischemia in a subset of patients. Elevated troponin levels can be due to a wide array of mechanisms in the absence of myocardial ischemia and injury. Thus, relying solely on troponin levels, in the presence of a normal electrocardiogram (ECG), to diagnose myocardial ischemia can lead to unnecessary and expensive invasive testing. It is therefore important for the clinician to keep in mind the varying causes of troponin elevations in order to provide the highest value care to the patient. We present a case and review of literature regarding patients who present with elevated troponin levels in the absence of any coronary artery disease.

## Introduction

Cardiac enzymes coupled with electrocardiogram (ECG) and a clinical picture of chest pain have traditionally been the trifecta used in the diagnosis of acute myocardial infarction (MI). However, it is becoming increasingly more frequent to see patients with little to no symptoms and/or ECG changes, requiring more reliance upon cardiac enzymes as the tool of choice to diagnose non-ST elevation myocardial infarction (NSTEMI) [1, 2].

It is important to note that although MI may be the most common cause of elevated cardiac enzymes, more specifically troponin, it is certainly not the only cause [1, 2]. The elevated troponins in the absence of myocardial ischemia are rare; however, it can be seen in conditions such as myocarditis, pulmonary embolism, acute heart failure, sepsis and septic shock, hypovolemia, renal failure, atrial fibrillation and cardiac contusion [2, 3].

This paper discusses a case of a patient with an unexplained elevation of troponin I (cTnI) in the absence of coronary artery disease and other relatively common non-cardiac etiologies. The numerous causes of non-coronary-related cTnI elevations are discussed.

## Case Report

This is a case of a 57-year-old female with a past medical history of hypertension, dyslipidemia, depression, osteoarthritis and NSTEMI 2 years prior to admission, who recently traveled from Florida to New Jersey 3 weeks prior to admission, and had received a corticosteroid injection into her knee for the treatment of osteoarthritis earlier the day of admission.

This patient came to the emergency department complaining of palpitations and lightheadedness. She denied any shortness of breath, chest pain, diaphoresis or nausea and/or vomiting. She denied any sick contacts, fever, chills, cough or any recent flu-like symptoms.

On further evaluation of this patient, ECG showed sinus tachycardia with HR of 102/min without any ST-T deviation, and chest X-ray showed no acute process. NT Pro-BNP was found to be 61.75 pg/mL (significant if > 450 pg/mL in those < 50 years of age, > 900 pg/mL in those 50 - 75 years of age, > 1,800 pg/mL in those > 75 years of age, if < 300 heart failure is highly unlikely) and a D-dimer of 0.23 μg/mL (normal value < 0.50 μg/mL); further laboratory tests including TSH and rheumatoid factor were within normal limits, except for a cTnI of 4.01 ng/mL (normal value 0 - 0.1 ng/mL).

CT angiogram of the chest revealed no pulmonary embolism. An echocardiogram was done to assess the cardiac function of the patient and revealed a left ventricle with normal structure and function, no wall-motion abnormalities, no dilatation of the ventricle and an ejection fraction > 55%, without any evidence of pulmonary hypertension, pericardial effusion or aortic dissection.

During this time, the patient’s cTnI continued to increase and peaked at 4.95 ng/mL (normal 0 - 0.1 ng/mL), and a cardiac catheterization was done, which showed normal coronary arteries without any evidence of coronary artery disease, no hypokinesis of the ventricular walls, no ballooning of the apex, and an intra-catheterization LVEF of 65%.

It is interesting to note that this patient had a similar episode 2 years prior to this admission, which was her “NSTEMI”, where the patient’s presenting cTnI level was 2.26 ng/mL (normal 0 - 0.1 ng/mL), and again a full cardiac workup was done, including echocardiogram and cardiac catheterization, demonstrating normal cardiac function without any evidence of coronary artery disease, hypokinesis of the left ventricle, and no evidence of either systolic or diastolic left ventricular dysfunction by echocardiography.

The patient remained stable and chest-pain free post cardiac catheterization and was discharged home shortly thereafter on lisinopril and aspirin. Curiously, both times the patient presented with elevated troponins, and she was discharged with elevated troponins and no definite diagnosis; however, we theorize that, given her history of depression, she may have been suffering from a milder apical-sparing variant of stress cardiomyopathy [4, 5].

## Discussion

It is well known that cardiac enzymes, more specifically troponin, play a vital role in the diagnosis of acute coronary syndrome (ACS) and possible an MI, especially in the setting of ambiguous symptology or a non-specific ECG [1]. The release of troponins is predominantly controlled by irreversible myocyte damage and subsequent myocyte necrosis [6]; however, it is also known that cardiac troponins may be released into the blood stream in the absence of myocyte damage/necrosis [6].

There are three types of troponins that are released into the human blood stream, troponin C, troponin T (cTnT), and cTnI, each having a unique function [5]. However, cTnT and cTnI are expressed by cardiac muscle, and therefore the preferred troponin for measurement in myocardial injury [7].

In 2012, the Multinational Third Global MI Task Force updated the universal definition of MI to include the detection of cardiac biomarkers, one of which was elevated, preferably cTn [1]. Furthermore, patients must have at least one of five diagnostic criteria (Table 1) [1]. In addition to this new definition, the 2012 Task Force further evaluated and outlined the multiple mechanisms and/or types of MI (Table 2) [1].

**Table 1:** Additional Diagnostic Criteria (Apart From Cardiac Biomarkers) for the Diagnosis of Myocardial Infarction as per the 2012 Joint ESC/ACCF/AHA/WHF Task Force for the Universal Definition of Myocardial Infarction [1]

Symptoms of ischemia
New significant ST/T wave changes or new left bundle branch block on ECG
Pathological Q waves on ECG
Imaging evidence of myocardial wall motion abnormalities
Intra-catheterization or post-mortem evidence of coronary thrombus

**Table 2:** Types/Mechanism of Myocardial Infarction [1]

I	Primary myocardial ischemia, i.e. plaque rupture, thrombus formation
II	Supply/demand ischemia, i.e. vasospasm, hypotension, arrhythmia, severe anemia
III	Injury without ischemia, i.e. myocarditis, cardiac contusion
IV	Multifactorial, i.e. heart failure, renal failure

The causes of myocardial damage range from coronary to non-coronary etiologies. Coronary causes of myocardial ischemia include ACS, cocaine use, coronary intervention, coronary artery spasm, severe hypertension, heart failure, acute aortic dissection and coronary artery vasculitis [2, 8]. However, for the purposes of this paper, the focus will be maintained on non-coronary causes of troponin elevations.

There are multiple causes of elevated troponins in the absence of ACS, which Thygesen et al have conveniently grouped into three major categories: 1) myocardial damage due to myocardial ischemia [8]; 2) myocardial damage due to non-ischemic causes [8]; 3) idiopathic myocardial injury [8].

In the setting of non-coronary myocardial ischemia, common causes include sepsis and septic shock, hypoxia, hypo-perfusion, and pulmonary embolism (Table 3) [2, 8-12].

**Table 3:** Causes (Non-Acute Coronary Syndrome) of Elevated Troponin by System [9-12]

Cardiovascular	Respiratory
Aortic dissection	Pulmonary embolism
Arrhythmia	ARDS
Heart failure	Infectious/immune
Endocarditis/myocarditis/pericarditis	Severe sepsis/septic shock
LVH	Critical illness
Infiltrative disease	TTP
Stress cardiomyopathy	Gastrointestinal
Mechanical injury	Severe GI bleeding
Blunt trauma/contusion	CNS
Cardiac surgery/angioplasty/ablation/cardioversion	Ischemic/hemorrhagic stroke
CPR	Severe head trauma
Musculoskeletal/skin	Renal
Rhabdomyolysis	Chronic kidney disease
Extensive burns	Other
Toxins/drugs	Strenuous exercise
Cocaine	
Cardiotoxic chemotherapy	
Cyanide intoxication	
Carbon monoxide poisoning	
Amphetamines	
Scorpion/snake/box jellyfish venom	

Many studies have shown the correlation of elevated troponins and the severity of sepsis, and found it to correlate with the degree of hypotension and the APACHE II score [10]. Many authors have hypothesized that severe sepsis may induce the release of myocardial depressive factors that lead to the breakdown of larger free troponin to smaller, lower-molecular-weight fragments, which, through increased membrane permeability, make their way into systemic circulation [11]. However, other theories suggest circulating cytokines as well as bacterial endotoxins cause a direct toxic effect on the myocardium, thus inducing the release of troponins [8]. However, ultimately, the exact mechanism remains unclear.

While in pulmonary embolism, elevated troponins are associated with a worse prognosis. In a meta-analysis of multiple pulmonary embolism studies, patients with elevated troponin levels had greater than five times the mortality of those with normal troponin levels [11]. The mechanism behind troponin release in these patients is thought to be related to right ventricular dilatation and strain from increased pulmonary arterial resistance [10].

Additionally, there are multiple causes of elevated troponins and myocardial damage in which ischemia is not involved, and these include renal failure, infiltrative diseases, extreme exertion, cardiac contusion, extensive burns, acute pericarditis, myocarditis and stress cardiomyopathy, among many others (Table 3) [2, 8-11].

In acute pericarditis, elevations of troponins are believed to be related to acute inflammation and subsequent injury to the epicardial layer of the myocardium, thus causing a release of troponins [10]. Another common cause of troponin elevations in the absence of myocardial ischemia is renal failure; although, usually it is the cTnT, not usually cTnI, that is elevated [2-4, 9-11]. While the exact cause of this elevation of troponins in renal failure remains unknown, clinical outcomes have shown a worse prognosis for those patients with elevated troponin levels [10].

Stress cardiomyopathy, also known as Takotsubo cardiomyopathy or broken-heart syndrome, is caused by intense emotional or physical stress leading to rapidly occurring and severe reversible cardiac dysfunction, that typically occurs in women, especially post-menopausal women, accounting for approximately 1.05-2.2% of patients presenting with symptoms originally thought to be ACS [11, 13]. Although the exact mechanism remains unclear, it has been hypothesized that an exaggerated sympathetic stimulation may lead to a catecholamine-induced direct myocyte injury [11]. Furthermore, there seems to be a higher prevalence of stress cardiomyopathy among those suffering from anxiety and depression. Ziegelstein had reported that patients with psychiatric disorders may have a stronger catecholamine response to stress, as well as a decreased inhibition of catecholamine release [14, 15].

Apical sparing variants of stress cardiomyopathy have been reported in the past, in which the apical ballooning was thought to be transient, and therefore not visualized on imaging and diagnostic studies [4, 5, 13]. Other authors hypothesize that the hypokinesis/akinesis may be mid-ventricular, sparing the apex [4, 5, 16].

There have been multiple accounts of false-positive troponin elevations. Factors that may interfere with the cTnI include heterophilic antibodies, rheumatoid factor, fibrin clots and equipment malfunction (Table 4) [12, 17, 18].

**Table 4:** Causes of False-Positive Troponin Elevations [12, 17]

Heterophilic antibodies
Rheumatoid factor
Fibrin clots
Microparticles in the specimen
Immunocomplex formation
Analyzer malfunction

Heterophilic antibody induced troponin elevation has been reported by multiple studies with an incidence ranging from 0.17% to 40% [17, 18]. These heterophilic antibodies are usually acquired from iatrogenic and non-iatrogenic causes, including transfusion of blood products, vaccinations, autoimmune diseases and monoclonal antibody therapies, to name a few [19].

Rheumatoid factor elevations can cause falsely elevated cTnI levels as well. Krahn et al report that 5% of healthy patients have circulating rheumatoid factor, and an estimated 1% of patients with elevated cTnI levels have this elevation solely due to circulating rheumatoid factor [20, 21]. Furthermore, a recent study of patients with elevated cTnI levels with rheumatoid factor found that 11.5% were heterophilic false positive [20], and even after pre-treatment with heterophile blocking agents, approximately 50% of the false positives were not corrected [20].

Finally, idiopathic myocardial injury has been found to be a much more common phenomenon than previously believed. A study done by Bakshi et al, of 21 patients with elevated cTnI levels with normal coronary arteries on catheterization, was done to determine the etiology of the elevated troponin levels [20]. This study revealed the most common etiology was “no clear precipitating event” accounting for 47% of the patients [20]. Additional studies done by Assomull et al were done to determine the cause of elevated troponin levels in non-obstructive coronary artery disease, which revealed of their patient population, 50% had elevated troponin levels due to myocarditis [21], only 11.6% of these patients had elevated troponins due to an actual myocardial infarction [21], and 35% of these patients had elevated troponin levels with no clear diagnosis [21].

In our case, patient had elevated troponins on two separate occasions 2 years apart. However, defining our patient’s condition, and those with similar circumstances, as “idiopathic” simply means that there are unknown causes of troponin elevations which require further clinical and molecular investigation in the future. One of these possibilities may be that of a milder apical-sparing variant of stress cardiomyopathy, leading to myocyte injury without hypokinetic changes [4, 5].

### Conclusion

While troponin plays a vital role in the diagnosis of NSTEMIs, it is imperative to keep in mind the multiple non-ischemic and non-cardiac causes and potential factors that may lead to elevations of troponin levels.

The most common causes of elevated troponins can be divided into ACS and non-ACS causes. The most common ACS causes are comprised of STEMI, NSTEMI and unstable angina, generally speaking. While the non-ACS cause list is much longer, the most common causes include pulmonary embolism, sepsis and septic shock, pericarditis, myocarditis, cocaine use, aortic dissection, CKD and coronary artery spasm.

Additionally, this patient had a negative rheumatoid factor, although heterophile antibody was never tested on this patient, it is unlikely that on two separate occasions this patients had false positive troponin elevations. Furthermore, elevation of her troponin levels 2 years apart makes equipment malfunction unlikely.

In the case of our patient, we are hypothesizing that our patient, who is a middle-aged, post-menopausal female with a history of depression, may have suffered a milder apical-sparing variant of stress cardiomyopathy without any hypokinetic changes on diagnostic imaging [4, 5].

## Abbreviations

NSTEMI: non-ST elevation myocardial infarction
MI: myocardial infarction
cTnI: troponin I
ECG: electrocardiogram
cTnT: troponin T
ACS: acute coronary syndrome
